# Endovascular treatment of *Brucella*-infected abdominal aortic aneurysm: A case report

**DOI:** 10.1097/MD.0000000000007666

**Published:** 2017-10-20

**Authors:** Tao Zhang, Donghua Ji, Feng Wang

**Affiliations:** Department of Interventional Therapy, First Affiliated Hospital of Dalian Medical University, Dalian, China.

**Keywords:** *Brucella*, computed tomography angiography, endovascular, endovascular aneurysm repair, infected abdominal aortic aneurysm

## Abstract

**Rationale:**

In very rare cases, a primary infected abdominal aortic aneurysm (IAAA) is caused by a species of *Brucella*. In this report, we report such a case that was successfully treated with a novel approach. To the best of our knowledge, this was the first case occurring in China, in which an infection of the abdominal aortic aneurysm was caused by a *Brucella* species.

**Patient concerns:**

The clinical findings included high fever, fatigue, and abdominal pain.

**Diagnoses:**

The diagnosis was confirmed by computed tomography angiography and by bacteriologic isolation from the patient's blood culture.

**Interventions:**

The patient was given endovascular aneurysm repair (EVAR) and *Brucella*-sensitive antibiotics for 6 weeks.

**Outcomes:**

During the 10-month follow-up, the patient's clinical course remained uneventful.

**Lessons:**

Our case study supports the premise that endovascular aneurysm repair is an appropriate alternative strategy to treat an infected abdominal aortic aneurysm. Compared with conventional surgical treatment, EVAR with long-term oral antibiotics is a simpler, less traumatic, and more efficient procedure. However, this needs to be further evaluated through long-term follow-up.

## 1 Introduction

*Brucella* is a genus of aerobic gram-negative bacteria. These intracellular pathogens are immobile, and nonencapsulated. *Brucella* can survive in vivo in several animals, including sheep and cattle. In humans, *Brucella* causes a disease named Malta fever (i.e., Brucellosis). Thus, *Brucella* is considered a zoonotic pathogen. The people most susceptible to be attacked from *Brucella* are those who are in direct contact with infected animals.

In extremely rare cases, *Brucella* causes primary infected abdominal aortic aneurysm (IAAA). Only 1 such case has been reported in Korea. In China, no case of primary infection by *Brucella* has been reported previously. Herein, we describe a case of primary IAAA caused by *Brucella* and discuss its clinical manifestations and diagnosis. We performed endovascular aneurysm repair (EVAR) to treat this exceptionally rare case. The patient consented to the publication of this report.

## 2 Case report

A 63-year-old man experienced intermittent abdominal pain with fever for more than 10 days, a high temperature reaching 39 °C. However, in hospital, he had normal blood pressure.

**Figure 1:**
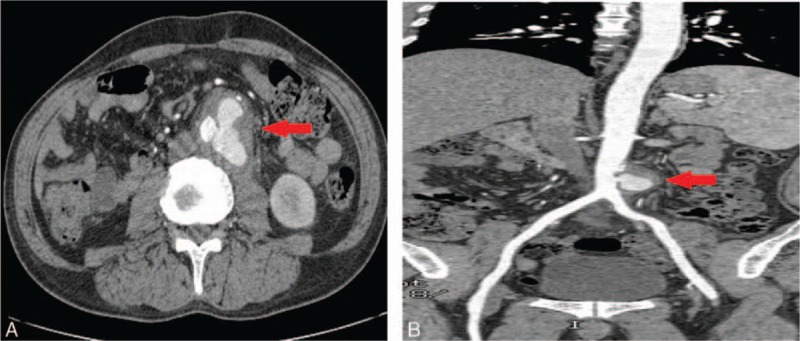
An abdominal aortic pseudoaneurysm observed on 3D-CTA (A and B, arrows). 3D-CTA = three-dimensional computed tomography angiography.

A physical examination revealed a palpable, pulsatile mass in the left abdomen. By performing computed tomography angiography (CTA), we further confirmed the presence of an abdominal aortic pseudoaneurysm (Fig. 1), which was surrounded by tissues. The likelihood of rupture associated with this aneurysm was high, due to the irregular shape of the aneurysm and the persistent abdominal pain of the patient.

Blood test results were as follows: peripheral white blood cells 3.03 × 10^9^/L; C-reactive protein 58.1 mg/L; and erythrocyte sedimentation rate 24 mm/h. Transthoracic echocardiogram revealed no evidence of endocarditis or vegetation. Based on the clinical manifestations, physical signs, and results of biochemical tests, we suspected this to be a case of infected abdominal aortic aneurysm.

**Figure 2:**
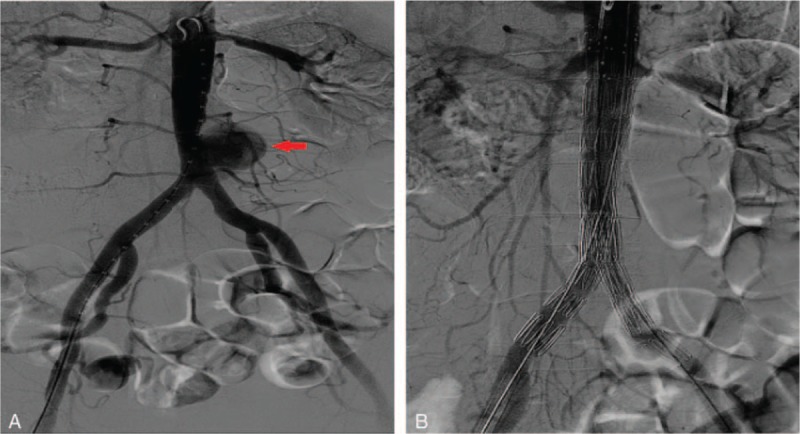
Images of abdominal aortic pseudoaneurysm before and after endovascular treatment. (A) Digital subtraction angiography clearly shows an abdominal aortic pseudoaneurysm. (B) After performing EVAR, the abdominal aortic pseudoaneurysm was completely isolated. Arrows indicate the abdominal aortic aneurysm.

To tackle this persistent infection, we administered the antibiotic cefoperazone sulbactam. Then, we used a blood culture to determine the pathogen that caused this infection. To prevent the rupture of this aneurysm, we performed percutaneous EVAR with the patient under local anesthesia. Abdominal aortic angiography revealed that the pseudoaneurysm was in the lower segment of the abdominal aorta. Its size was about 5.2 cm × 3.0 cm, and distal bifurcation was not significantly involved (Fig. 2). A blood sample (20 mL) was collected from the internal arterial aneurysm to prepare a blood culture. A Zenith Flex graft main body of 26 mm × 96 mm (Cook, Bloomington) was passed through the abdominal aorta to block completely the aneurysm. A Zenith iliac branch (16 mm × 39 mm; Cook, Bloomington) was placed at the left external iliac artery distal end, closing the entrance of the left internal iliac artery—this occurred by chance, because only the shortest iliac graft with a length of 39 mm was at hand. Another Zenith iliac branch (16 mm × 56 mm) was placed in the contralateral. However, the entrance of the distal end of the right internal iliac artery was left open. A postoperative angiography confirmed a complete closure of the aneurysm cavity (Fig. 2).

After the operation, levofloxacin and vancomycin were administered, as they are broad-spectrum antibiotics that can tackle any persistent infection. A *Brucella* antibody test of the blood culture obtained from both the arterial aneurysm and the peripheral circulatory system (3×) indicated *Brucella* infection.

When we asked about the patient's daily activity, he told us he raised a large number of goats, indicating that he was in direct contact with infected animals. This was a major factor that promoted development of *Brucella* infection in the patient. As a precautionary measure, we then changed the course of antibiotics and prescribed a combination of doxycycline 100 mg twice daily, and rifampicin 600 mg once daily, which were administered orally to the patient. One week after the operation, the patient had attained a normal body temperature and did not exhibit any abnormal changes in kidney function. Furthermore, he did not experience any abdominal pain, back pain, or other symptoms.

**Figure 3:**
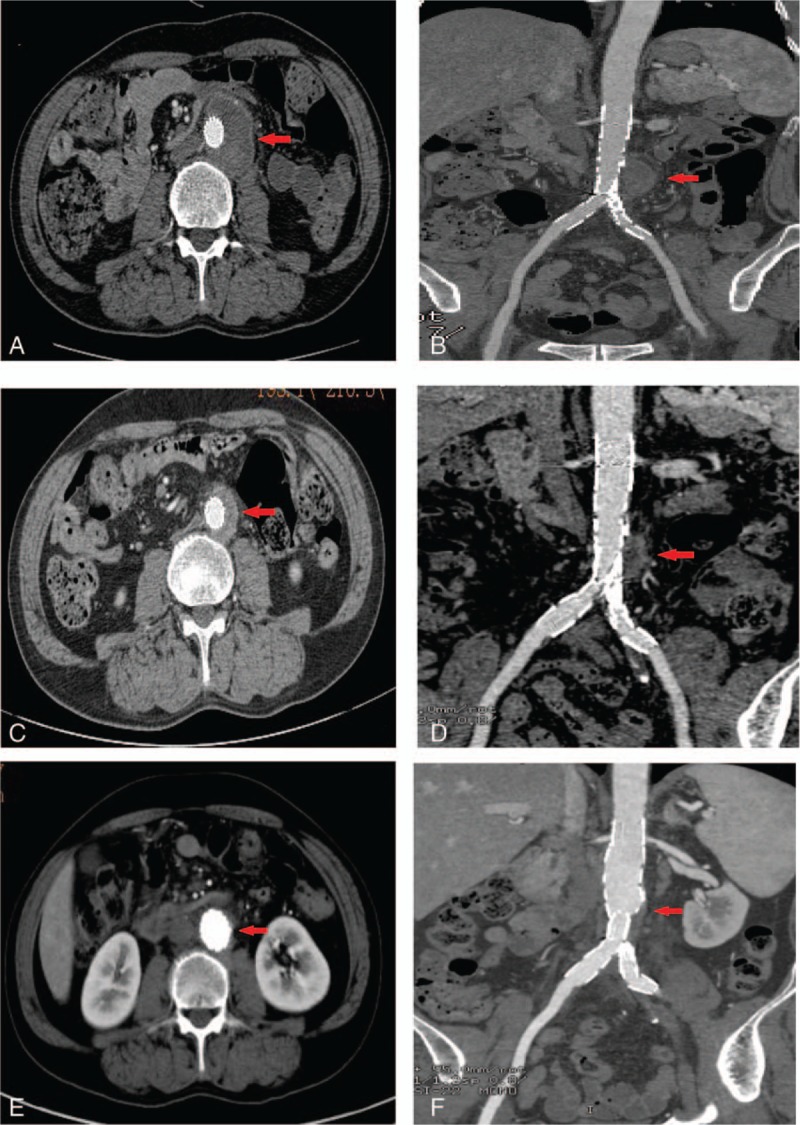
Three-dimensional CTA imaging shows complete isolation and shrinking of the abdominal aortic pseudoaneurysm. (A, B) One week after performing endovascular repair, there was a complete isolation of the abdominal aortic pseudoaneurysm with no signs of an endoleak. (C, D) Fifty days after the operation, the abdominal aortic pseudoaneurysm had shrunk significantly. There were no signs of infection in the surrounding tissues. (E, F) Ten months after the operation, the abdominal aortic pseudoaneurysm had disappeared almost completely. Arrows indicate the original position of the abdominal aortic aneurysm. 3D-CTA = three-dimensional computed tomography angiography.

CTA imaging revealed a satisfactory isolation of the aneurysm (Fig. 3A and B). However, the oral administration of doxycycline 100 mg twice daily and rifampin 600 mg once daily was continued for 6 weeks postoperatively. After 50 days’ follow-up, the patient had no fever or abdominal pain. Furthermore, the liver and kidney function of this patient were also normal. CTA imaging displayed a complete closure of the original abdominal aortic pseudoaneurysm cavity, and there were no signs of an endoleak from this cavity. The size of the tissues surrounding the aneurysm had also shrunk significantly (Fig. 3C and D).

At the 10-month follow-up after the operation, the abdominal aneurysm had disappeared almost completely (Fig. 3E and F). The white blood cells and erythrocyte sedimentation rate were normal, with negative blood cultures.

## 3 Discussion

IAAA is a rare, life-threatening disease. Patients with IAAA often die of aneurysm rupture or sepsis.^[1]^ To treat IAAA, surgeons perform traditional surgical interventions, such as surgical aneurysm resection, reconstruction of local anatomical structures via in situ revascularization or bypass extra-anatomic reconstruction, foci debridement, and adequate drainage.^[2]^ However, traditional open surgery involves complex techniques and procedures, including a complete exposure of the infrarenal aorta, aneurysm, and iliac arteries. Therefore, this surgical intervention causes trauma and is associated with high morbidity and mortality.^[2,3]^

In contrast, endovascular treatment of aortic aneurysm infection has been gaining attention in recent years.^[4]^ We assume that as long as the vascular anatomic conditions meet the requirements of a standard EVAR treatment, endovascular treatment may be considered, as it has high efficacy.^[5–8]^

In general, the most common pathogens that cause IAAA are *Staphylococcus aureus* (40%), *Salmonella* (15%), *Streptococci* (8%), and *Escherichia coli* (7%), and in some cases *Staphylococcus epidermidis*, *Klebsiella*, *Haemophilus influenzae*, and *Mycobacterium tuberculosis*.^[1]^ In humans, *Brucella* mainly affects the motor, respiratory, digestive, urinary, and reproductive systems. However, infection of the cardiovascular system by *Brucella* is uncommon, and it is even rarer that *Brucella* infection directly causes aortic aneurysm, which is usually secondary to endocarditis in clinic. In 2007, Park et al^[9]^ reported the world's first *Brucella*-infected abdominal aortic aneurysm in South Korea.

In the present report, IAAA caused by *Brucella* was diagnosed in our patient; the findings were confirmed by CTA imaging, clinical manifestations, the blood culture, and the daily contact with infected livestock. Postoperatively, the patient was prescribed doxycycline and rifampin, which are very strong antibiotics that effectively combat *Brucella*. In a previous case of *Brucella*-IAAA (2007), the aneurysm was surgically resected and revascularized to treat the patient. However, in the present case, we performed standard EVAR to treat the IAAA of this patient, based on the anatomical features of the pseudoaneurysm and the patient's basic condition. To avoid the trauma of an open surgery and the risk of mortality, we performed the standard EVAR procedure under local anesthesia and achieved appreciable results. During the 10 months’ follow-up, the patient did not report any clinical manifestations, which was also supported by CTA imaging. According to our communicable disease specialist's suggestion, antibiotic treatment for this patient lasted 50 days postoperatively, but not lifelong to avoid the adverse effects of doxycycline and rifampin on liver and kidney function. Until the present writing, we have not seen any sign of graft infection.

Based on the clinical results of this patient, we propose that EVAR can be used to treat selected patients with IAAA.^[10]^ Compared to the conventional surgical treatment, EVAR with long-term oral antibiotics is a simpler, less traumatic and more efficient procedure. However, this needs to be further evaluated through long-term follow-up.^[11]^
